# Expression of Long Non-Coding RNAs by Human Retinal Müller Glial Cells Infected with Clonal and Exotic Virulent *Toxoplasma gondii*

**DOI:** 10.3390/ncrna5040048

**Published:** 2019-09-20

**Authors:** Elise Rochet, Binoy Appukuttan, Yuefang Ma, Liam M. Ashander, Justine R. Smith

**Affiliations:** Flinders University College of Medicine & Public Health, Adelaide, SA 5042, Australia; rochet.elise@gmail.com (E.R.); binoy.appukuttan@flinders.edu.au (B.A.); yuefang.ma@flinders.edu.au (Y.M.); liam.ashander@flinders.edu.au (L.M.A.)

**Keywords:** *Toxoplasma gondii*, toxoplasmosis, human, eye, retina, Müller cells, long non-coding RNA

## Abstract

Retinal infection with *Toxoplasma gondii*—ocular toxoplasmosis—is a common cause of vision impairment worldwide. Pathology combines parasite-induced retinal cell death and reactive intraocular inflammation. Müller glial cells, which represent the supporting cell population of the retina, are relatively susceptible to infection with *T. gondii*. We investigated expression of long non-coding RNAs (lncRNAs) with immunologic regulatory activity in Müller cells infected with virulent *T. gondii* strains—GT1 (haplogroup 1, type I) and GPHT (haplogroup 6). We first confirmed expression of 33 lncRNA in primary cell isolates. MIO-M1 human retinal Müller cell monolayers were infected with *T. gondii* tachyzoites (multiplicity of infection = 5) and harvested at 4, 12, 24, and 36 h post-infection, with infection being tracked by the expression of parasite surface antigen 1 (SAG1). Significant fold-changes were observed for 31 lncRNAs at one or more time intervals. Similar changes between strains were measured for BANCR, CYTOR, FOXD3-AS1, GAS5, GSTT1-AS1, LINC-ROR, LUCAT1, MALAT1, MIR22HG, MIR143HG, PVT1, RMRP, SNHG15, and SOCS2-AS1. Changes differing between strains were measured for APTR, FIRRE, HOTAIR, HOXD-AS1, KCNQ1OT1, LINC00968, LINC01105, lnc-SGK1, MEG3, MHRT, MIAT, MIR17HG, MIR155HG, NEAT1, NeST, NRON, and PACER. Our findings suggest roles for lncRNAs in regulating retinal Müller cell immune responses to *T. gondii*, and encourage future studies on lncRNA as biomarkers and/or drug targets in ocular toxoplasmosis.

## 1. Introduction

*Toxoplasma gondii* is a ubiquitously distributed protozoan parasite that may infect any warm blooded animal [1]. Clinical disease in humans is related to localization to the central nervous system. Ocular toxoplasmosis follows the entry of *T. gondii* into and replication within the retina, which is associated with a vigorous inflammatory reaction; the inflammation may reach other ocular tissues, such as choroid, vitreous, or more rarely the optic nerve [1,2]. Ultimately the reaction resolves, but leaves scars in the retina that are associated with the parasite encystment. Subsequent rupture of a retinal cyst leads to the reactivation of the condition, followed by a new attack of inflammation. Although ocular toxoplasmosis is a worldwide disease, differences in clinical manifestations are observed, and are related to the host genetics, geographical region, and parasite genotype [3,4,5]. In Western Europe and North America, where 2% of the *T. gondii* seropositive population have retinal lesions, most of the parasite strains belong to type I, II, and III, and haplogroup 12 clonal lineages [6,7]. On the contrary, in South America, many non-clonal haplogroups are found [8,9,10], which are linked to more severe presentations and a higher prevalence of retinal lesions, reaching as high as 17% [11,12,13].

Müller cells are a unique retinal cell population with multiple biological functions [14]. They are the predominant glial cell of the eye, extending between the internal and external limiting membranes of the retina and providing support for all neurons. Müller cells are a first line of defense during retinal infection, and are involved in the innate immune response to a pathogen by producing cytokines and chemokines necessary to attract and activate leukocytes [15,16,17]. However, they are unable to control tachyzoite proliferation alone [18]. Müller cells also are a preferential target of *T. gondii* in the human retina—Furtado et al. [19] highlighted that Müller cells are more susceptible to the infection than retinal neurons. Along with ganglion neuronal cells, Müller cells are the intraocular cell population that harbors the latent form of the parasite—the bradyzoite [20,21,22,23].

Gene expression in both healthy and pathological microenvironments is directed in part by a diverse group of regulatory long non-coding RNAs (lncRNAs) [24]. Long non-coding RNAs are defined by lack of protein-coding ability and a length greater than 200 nucleotides. However, recent studies have demonstrated that some lncRNAs encode micropeptides with possible biological activity [25,26]. Most lncRNAs are transcribed by RNA polymerase II, and may be spliced, polyadenylated, and 5′ capped. Conservation across species is generally poor, and lncRNAs usually have low abundance in cells, with a few exceptions, such as metastasis associated lung adenocarcinoma transcript 1 (MALAT1), nuclear paraspeckle assembly transcript 1 (NEAT1), and homeobox gene transcript antisense RNA (HOTAIR) [27]. Expression is cell and/or tissue-specific, and may vary over time and in response to local molecular conditions. According to cellular localization, and secondary and tertiary structures, lncRNAs may bind DNA, RNA, and protein to influence molecular functions in *cis* or *trans* [28,29]. As regulators of gene expression, lncRNAs participate in a wide range of biological processes [30,31], acting in multiple overlapping molecular roles, including as signalers, enhancers, scaffolds, decoys, and guides [32]. Dysregulation of lncRNAs has been connected to a variety of pathologies, such as cancers, immune and infectious diseases, neurological conditions, and genetic disorders [30,33].

During infection, host lncRNA expression in myeloid and non-myeloid cells may change, consistent with a role in the regulation of host–pathogen interactions and the outcome of infection [28]. Certain lncRNAs promote pathogen clearance (e.g., host nettoie salmonella pas Theiler’s (NeST) in *Salmonella enterica* infection) [34], whereas others facilitate survival of the pathogen (e.g., host non-coding repressor of nuclear factor of activated T-cells (NRON) during human immunodeficiency virus infection) [35]. The involvement of lncRNAs in *T. gondii* infection of Müller cells is unstudied. Indeed, there is little published literature on the role of Müller cells during a *T. gondii* infection [18,19,36], or during intracellular pathogen infection in general [37,38]. Here, we were interested in the lncRNA response of Müller cells to infection with virulent strains of *T. gondii*, with a focus on the acute immune response. We first examined expression of 35 human lncRNAs with known immunologic activities in primary retinal Müller cells and the MIO-M1 cell line. Then, we used reverse transcription real-time quantitative polymerase chain reaction (RT-qPCR) to follow expressed lncRNAs over time in MIO-M1 cells infected with two natural virulent strains of *T. gondii*—GT1 (type I, haplogroup 1, common in the United States and Western Europe) and GPHT (haplogroup 6, common in South America).

## 2. Results

### 2.1. Expression of Long Non-Coding RNA in Human Retinal Müller Cells

We selected 35 lncRNAs for investigation—these lncRNAs were identified on the basis of a literature search directed at human lncRNA with role(s) in the immune responses. Prior to investigating the potential involvement of retinal Müller cell lncRNAs in the immune response to *T. gondii*, we first assessed expression of these 35 lncRNAs by cells under baseline conditions. We studied primary Müller cells, isolated from three cadaveric human donors, and the MIO-M1 human Müller cell line. Because of the large number of cells needed for the infectivity studies, it was necessary to use a cell line, and, thus, this first stage of our work served to confirm that the lncRNAs to be studied in the MIO-M1 cell line were also expressed by primary Müller cells.

**Figure 1:**
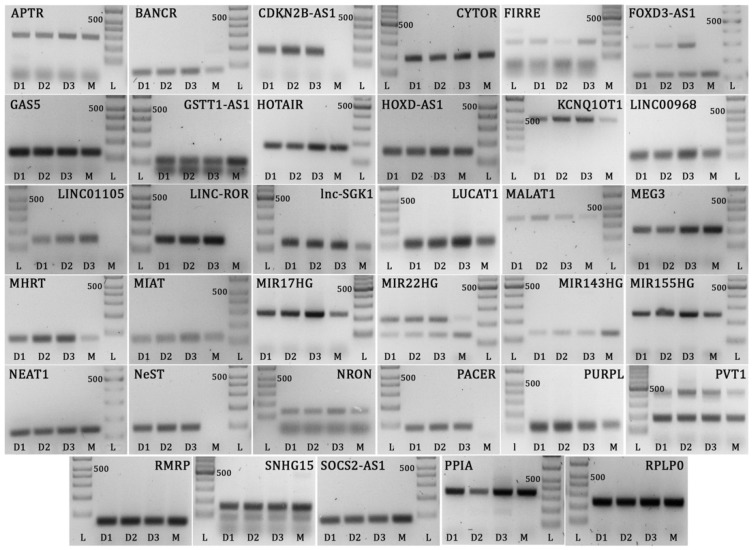
Gel images showing Müller cell long non-coding RNAs and reference gene amplicons run on 2% or 3% agarose gel. L = DNA ladder, D1 = human donor cell isolate 1, D2 = human donor cell isolate 2, D3 = human donor cell isolate 3, M = MIO-M1 cell line. Expected product sizes: APTR = 300 bp, BANCR = 80 bp, CDKN2B-AS1 = 169 bp, CYTOR = 187 bp, FIRRE = 215 bp, FOXD3-AS1 = 118 bp, GAS5 = 127 bp, GSTT1-AS1 = 105 bp, HOTAIR = 168 bp, HOXD-AS1 = 149 bp, KCNQ1OT1 = 567 bp, LINC00968 = 126 bp, LINC01105 = 137 bp, LINC-ROR = 151 bp, Lnc-SGK1 = 131 bp, LUCAT1 = 114 bp, MALAT1 = 396 bp, MEG3 = 187 bp, MHRT = 73 bp, MIAT = 122 bp, MIR17HG = 221 bp, MIR22HG = 136/219 bp, MIR143HG = 128 bp, MIR155HG = 247 bp, NEAT1 = 137 bp, NeST = 94 bp, NRON = 133 bp, PACER = 90 bp, PURPL = 120 bp, PVT1 = 179 bp, RMRP = 67 bp, SNHG15 = 155 bp, SOCS2-AS1 = 83 bp, PPIA = 355 bp, RPLP0 = 235 bp. Multiple bands on same gel (FOXD3-AS1, GSTT1-AS1, MIR22HG, PVT1, and SNHG15) represent transcript variants, which were confirmed by sequencing. Bands at lower edge of gels represent primer dimer products. Controls prepared with no template did not amplify.

Twenty-eight of the 35 selected lncRNAs were expressed in both primary Müller cells and the MIO-M1 Müller cell line (Figure 1): alu-mediated P21 transcriptional regulator (APTR), B-Raf proto-oncogene, serine/threonine kinase-activated non-protein coding RNA (BANCR), cytoskeleton regulator RNA (CYTOR or LINC00152), functional intergenic repeating RNA element (FIRRE), forkhead box D3-antisense RNA 1 (FOXD3-AS1), growth arrest specific 5 (GAS5), glutathione S-transferase theta 1 antisense RNA 1 (GSTT1-AS1 or lncRNA-CD244), HOTAIR, homeobox D cluster antisense RNA 1 (HOXD-AS1), potassium voltage-gated channel subfamily Q member 1 opposite strand/antisense transcript 1 (KCNQ1OT1), long intergenic non-protein coding RNA 968 (LINC00968), long non-coding RNA serum and glucocorticoid-inducible kinase (lnc-SGK1), lung cancer associated transcript 1 (LUCAT1), MALAT1, maternally expressed 3 (MEG3), myosin heavy chain associated RNA transcripts (MHRT), myocardial infarction associated transcript (MIAT or GOMAFU), MIR17 host gene (MIR17HG), MIR22 host gene (MIR22HG), MIR143 host gene (MIR143HG or CARMN), MIR155 host gene (MIR155HG), NEAT1, NRON, P53 upregulated regulator of P53 levels (PURPL), plasmacytoma variant translocation 1 (PVT1), RNA component of mitochondrial RNA processing endoribonuclease (RMRP), small nucleolar RNA host gene 15 (SNHG15), and suppressor of cytokine signaling 2-antisense RNA 1 (SOCS2-AS1).

Five of the 35 selected lncRNAs that were detected in primary Müller cells were not expressed in the MIO-M1 Müller cell line under non-stimulated conditions (Figure 1): long intergenic non-protein coding RNA, regulator of reprogramming (LINC-ROR), LINC01105, nettoie salmonella pas Theiler’s (NeST), cyclin-dependent kinase inhibitor 2B-antisense RNA 1 (CDKN2B-AS1), and PTGS2 antisense NFKB1 complex-mediated expression regulator RNA (PACER). The larger of the two FOXD3-AS1 transcripts was also not detected in MIO-M1 cells. Two of the 35 selected lncRNAs—LINC00305 and negative regulator of interferon response (NRIR or lncRNA-CMPK2)—were not expressed in primary cells or the cell line (data not shown), and therefore were not evaluated further in this work.

### 2.2. Time Course of T. gondii Growth in Human Retinal Müller Cells

**Figure 2:**
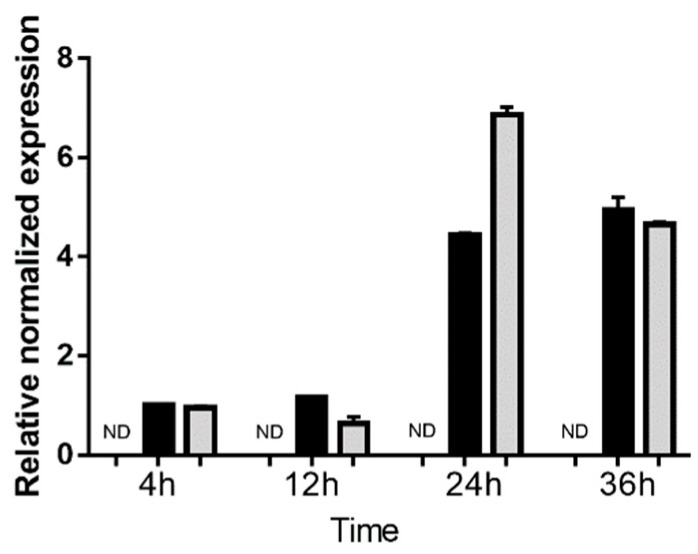
Expression of surface antigen 1 (SAG1) in human retinal Müller cells infected with *T. gondii* tachyzoites. Graph shows normalized SAG1 transcript expression in uninfected versus GT-1 and GPHT strain-infected MIO-M1 cells (multiplicity of infection = 5; evaluated time points post-infection = 4, 12, 24, and 36 h). Reference genes were ribosomal protein lateral stalk subunit P0 (RPLP0) and peptidylprolyl isomerase A (PPIA). *n* = 3 cultures/condition. Bars represent mean normalized expression, and error bars indicate standard deviation. Black columns represent GT1 strain-infected, and grey columns represent GPHT strain-infected.

Growth of GT1 and GPHT strain *T. gondii* tachyzoites in the MIO-M1 human retinal Müller cell line was assessed over 36 h by RT-qPCR for *T. gondii* surface antigen 1 (SAG1) (Figure 2). The level of SAG1 transcript was stable at low levels at 4 and 12 h post infection, consistent with invasion by the parasite. A marked increase in SAG1 expression was observed for cells infected with both strains at 24 h, indicating rapid intracellular replication of the parasite. By 36 h post-infection, there was no further increase in SAG1 expression, consistent with tachyzoite preparing to egress the cells, which typically occurs by approximately 48 h.

### 2.3. Expression of Long Non-Coding RNAs in Human Retinal Müller Cells during T. gondii Infection

**Figure 3:**
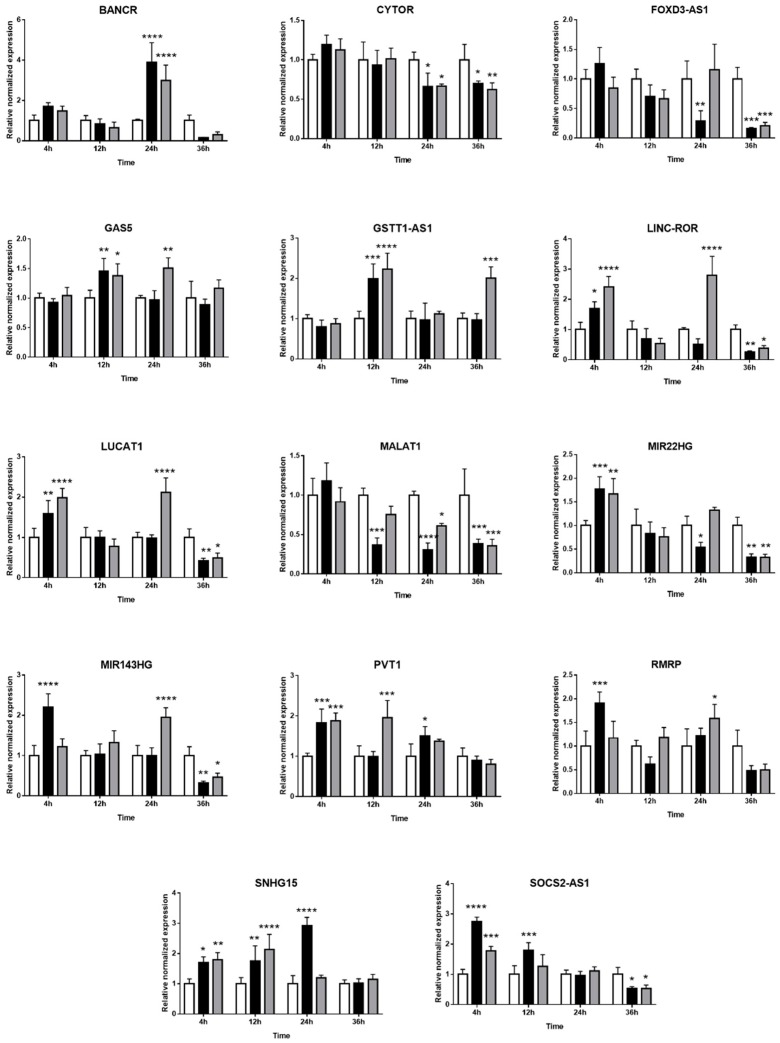
Expression of long non-coding RNAs in human retinal Müller cells infected with *T. gondii* tachyzoites with similarity in response to parasite strains. Graphs show normalized lncRNA expression in uninfected versus GT-1 and GPHT strain-infected MIO-M1 cells (multiplicity of infection = 5; evaluated time points post-infection = 4, 12, 24, and 36 h). Reference genes were ribosomal protein lateral stalk subunit P0 (RPLP0) and peptidylprolyl isomerase A (PPIA). Data were analyzed by two-way ANOVA with Bonferroni post-test. *n* = 3 cultures/condition. Bars represent mean normalized expression, and error bars indicate standard deviation. White columns represent uninfected, black columns represent GT1 strain-infected, and grey columns represent GPHT strain-infected. * *p* < 0.05, ** *p* < 0.01, *** *p* < 0.001, and **** *p* < 0.0001.

**Figure 4:**
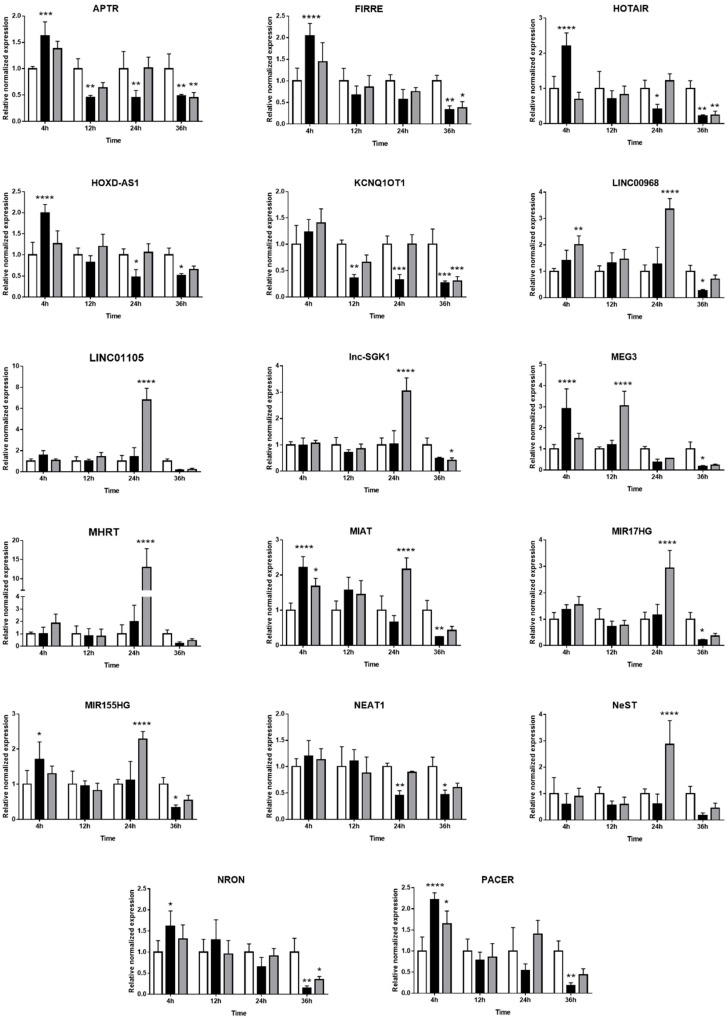
Expression of long non-coding RNAs in human retinal Müller cells infected with *T. gondii* tachyzoites with differences in response to parasite strains. Graphs show normalized lncRNA expression in uninfected versus GT-1 and GPHT strain-infected MIO-M1 cells (multiplicity of infection = 5; evaluated time points post-infection = 4, 12, 24, and 36 h). Reference genes were ribosomal protein lateral stalk subunit P0 (RPLP0) and peptidylprolyl isomerase A (PPIA). Data were analyzed by two-way ANOVA with Bonferroni post-test. *n* = 3 cultures/condition. Bars represent mean normalized expression, and error bars indicate standard deviation. White columns represent uninfected, black columns represent GT1 strain-infected, and grey columns represent GPHT strain-infected. * *p* < 0.05, ** *p* < 0.01, *** *p* < 0.001, and **** *p* < 0.0001.

The expression kinetics of 33 immune-related lncRNAs in Müller cells during *T. gondii* infection was evaluated by RT-qPCR in the MIO-M1 human retinal Müller cell line following infection with GT1 or GPHT strain tachyzoites. PURPL and CDKN2B-AS1 were not detected in these experiments (data not shown). For the remaining 31 lncRNAs, two patterns of expression were observed: (1) similarity in the changes in, or stability of, gene expression in infected versus uninfected Müller cells, when comparing infections by GT1 or GPHT strain tachyzoites (Figure 3), and (2) differences in the changes in, or stability of, gene expression in infected versus uninfected Müller cells, when comparing infections by GT1 or GPHT strain tachyzoites (Figure 4).

Among the common changes in lncRNAs that occurred in human retinal Müller cells infected with either *T. gondii* strain, as presented in Figure 3, some lncRNAs (i.e., LINC-ROR, LUCAT1, MIR22HG, MIR143HG, and SOCS2-AS1) were upregulated in the first 24 h post-infection as tachyzoites actively replicated, and downregulated at 36 h post-infection as tachyzoites prepared to egress. Other lncRNAs (i.e., BANCR, GAS5, GSTT1-AS1, PVT1, RMRP, and SNHG15) increased at one or more time-points during infection; BANCR was highly increased in infected cells, but only at 24 h. In contrast, CYTOR, FOXD3-AS1, and MALAT1 were consistently downregulated during the infection.

As presented in Figure 4, the two *T. gondii* strains influenced the expression of some lncRNAs in human retinal Müller cells in different ways. More lncRNAs were modulated during infection with GPHT—primarily manifested as high upregulation at 24 h—while infection with GT1 regulated the expression of lncRNAs in a more sustained manner. Thus, expression of LINC00968, LINC01105, lnc-SGK1, MHRT, MIR17HG, and NeST were only or largely impacted in GPHT infection. Some lncRNAs were differentially modulated depending on the parasite strain. APTR, FIRRE, HOTAIR, HOXD-AS1, MEG3, MIAT, MIR155HG, NRON, and PACER were upregulated at 4 h post-infection, and then immediately downregulated in GT1 infection, whereas they were only downregulated in GPHT infection at 36 h post-infection or not at all. LINC00968 was upregulated during the first 24 h of GPHT infection, but it was downregulated at 36 h after GT1 infection. NEAT1 and HOXD-AS1 expression were only modulated by the GT1 strain, whereas GPHT alone influenced LINC01105, lnc-SGK1, MHRT, and NeST expression.

At 36 h post-infection, consistent with imminent tachyzoite egress and Müller cell death, the majority of lncRNAs were downregulated in infected cells, with the exception of GSTT1-AS1, which was increased in GPHT-infected cells. Some lncRNAs were not significantly regulated in this final stage of the infection—in cells infected with both strains, BANCR, GAS5, LINC01105, MHRT, NeST, PVT1, RMRP, and SNHG15; in cells infected with GT1, GSTT1-AS1, and lnc-SGK1; and in cells infected with GPHT, LINC00968, MIAT, MIR17HG, HOXD-AS1, MIR155HG, NEAT1, and PACER.

## 3. Discussion

Little research has been published on the response of human retinal Müller cells to infection with *T. gondii*, although these cells are the key supporting cells within the retina, and ocular toxoplasmosis is a common retinal infection. We investigated the expression of lncRNAs in Müller cells infected with *T. gondii* tachyzoites to explore potential involvement of these regulatory molecules in ocular toxoplasmosis, focusing primarily on lncRNAs involved in the immune response and inflammation. We studied two virulent parasite strains for clear relevance to human disease—GT1 and GPHT. We measured significant changes at one or more time-points post-infection in the large majority of the lncRNAs that we studied (i.e., 31 of 35 lncRNAs). Although similarities in lncRNA expression were observed in both strains, more than half the lncRNAs studied were found to be differentially responsive to the strain. Interestingly, a few lncRNAs were modulated by one strain only—HOXD-AS1 and NEAT1 in GT1 infection, and LINC01105, lnc-SGK1, MHRT, and NeST in GPHT infection.

Long non-coding RNAs are involved in many aspects of the immune response, including immune homeostasis and inflammation, and the processes of leukocyte development, proliferation, and activation [39]. It is also clear that infectious pathogens may influence the expression of host lncRNA [28]. Using a microarray-based profiling approach, Liu et al. [37] and Menard et al. [38] independently evaluated the global lncRNA transcriptome in cells infected with *T. gondii* tachyzoites. There are many methodological differences between our investigation and these two studies, including molecular biological methods, strains of parasite, and host species and host cell population, all of which may impact experimental results. However, taking a discovery approach, both other groups found that the infection dysregulated substantial numbers of lncRNAs involved in the immune response. Liu et al. [37] used an array that covered 40,173 human lncRNAs to study infection of human neonatal fibroblasts by the type II avirulent strain ME49 strain—4525 lncRNAs were up-regulated, and 1519 lncRNAs were down-regulated, and coding-non-coding co-expression network analyses showed lncRNAs were involved in the expression of multiple immune-related proteins. Menard et al. [38] examined differential expression of lncRNAs in mouse bone marrow-derived macrophages infected with the type I virulent RH strain or the type II avirulent PTG strain—893 lncRNAs were up-regulated, and 875 lncRNAs were down-regulated, and gene ontology analyses demonstrated that these lncRNAs participated in many immune-related biological processes [38]. This second study highlighted a difference in the cellular response to the virulent and avirulent *T. gondii* strains, which was explained in part by a direct effect of the parasite ROP16 protein on host cell lncRNA expression [38].

In our results—obtained in human retinal Müller cells, which have direct relevance to ocular toxoplasmosis—LINC01105, BANCR, MIR17HG, MIR155HG, lnc-SGK1, MEG3, KCNQ1OT1, NEAT1, NeST, and MALAT1 were the most dysregulated lncRNAs with known immunologic activities. NeST is of particular interest, as it may act to boost a Th1 response by inducing IFN-γ transcription [34], and IFN-γ is critical to control the growth of *T. gondii* [40]. However, IFN-γ is produced primarily by activated lymphocytes [41], and a transcriptomic profiling study in progress indicates that human retinal Müller cells do not express this cytokine (data not shown). This lncRNA is transcribed anti-sense from a region of the genome that encodes interleukin (IL)-22 and IL-26, members of the IL-10 cytokine family, which may have anti-microbial activities [42]. The GPHT strain triggers earlier and higher expression of NeST than the GT1 strains, and this strain differential in the Müller cell immune response might have implications for clinical manifestations.

Other Müller cell lncRNAs that show significant changes in response to *T. gondii* infection—MEG3, MIR17HG and lnc-SGK1—are involved in regulation of the Th17 immune response [43,44,45,46]. This adaptive immune response, and production of IL-17 and IL-23 in particular, has been strongly implicated in the pathogenesis of ocular toxoplasmosis [47,48]. MEG3 is defined as a tumor suppressor, but also regulates the balance of Treg cells and Th17 cells; downregulation of MEG3 increases forkhead box P3 (FOXP3) expression and inhibits retinoic acid receptor-related orphan receptor γt (RORγt) expression, to tip the balance in favor of Treg cells [45,46]. On the other hand, MIR17HG and lnc-SGK1 promote the Th17 response [43,44]. Thus, our results suggest increased MEG3 may counterbalance the effects of increased MIR17HG and lnc-SGK1 in *T. gondii* infection. We also observed strong upregulation of BANCR in Müller cells at 24 h after infection with the virulent strains of *T. gondii* that we studied. An induction of BANCR by IL-13 has been demonstrated in vitro using a human esophageal epithelial cell line, ultimately leading to pro-inflammatory gene expression [49]. These molecular observations are in keeping with results of intraocular cytokinome profiling in South American patients, which identify IL-13 as a biomarker for severe ocular toxoplasmosis [5].

We also observed changes in several lncRNAs that have been implicated in cellular proliferation, invasion, and migration. LINC00968 has been studied mainly in cancer pathogenesis; it may promote tumor cell growth, migration, and invasion upon activation of Wnt/β-catenin or by targeting cyclin-A2 [50,51]. LINC00968 also may lead to apoptosis of the cell by recruiting enhancer of zeste homolog 2 (EZH2) to the promoter of p21, which inhibits p21 expression [52]. A critical role of BANCR in progression and metastasis of malignant melanoma and bronchial carcinoma is linked to the MAPK pathway [53,54]. On the other hand, LINC-ROR, first described as regulator of reprogramming [55], promotes c-Myc expression and interacts with p53 to generate an autoregulatory feedback loop that influences cell cycle progression and apoptosis [56]. *T. gondii* may have pro- or anti-proliferative effects, depending on the host cell population [57,58,59,60,61].

For this work on *T. gondii* infection, we employed the MIO-M1 human retinal Müller cell line in place of primary Müller cells. This was necessary, as we needed relatively large numbers of cells than could be generated reliably from individual paired human retinae. Freshly isolated human Müller cells are extremely slow growing. MIO-M1 cell line was spontaneously immortalized in 2002 from the eyes of a 68 year old female corneal donor [62], and is well characterized and widely used to study mechanisms of retinal disease. The MIO-M1 cell line provides reasonable transcriptomic fidelity to primary human Müller cells. As recently reported (bioRxiv unpublished preprint: https://doi.org/10.1101/425223), S.W. Lukowski et al. profiled the MIO-M1 cell line against other retinal cell types, and identified a glial cell transcriptome, with similarity to that of astrocytes, as well as Müller cells. Apart from CDKN2B-AS1, all the lncRNAs that we identified in primary Müller cells were also expressed by MIO-M1 cells, either at baseline or as a result of infection. However, alterations in the expression of a specific lncRNA of interest could be confirmed in primary Müller cells for the time-point at which we measured maximum dysregulation.

In conclusion, our work opens up a new area of research on the involvement of lncRNAs in the interaction between *T. gondii* and human retinal host cells, with implications for the pathogenesis—and ultimately the management—of ocular toxoplasmosis. Despite the fact that understanding of lncRNA activity in infectious diseases is still relatively limited, their major involvement as regulators of gene expression make lncRNAs attractive as therapeutic targets or biomarkers. Our work is limited to a descriptive in vitro study, without mechanistic experiments designed to test the roles of lncRNA in *T. gondii* infection in retinal cells in vitro or in ocular toxoplasmosis in vivo. We observed changes over the course of *T. gondii* infection of retinal Müller cells for almost all the lncRNAs that we studied. These changes were often impacted by parasite strain, and future work might be directed at evaluating other strains that may cause ocular toxoplasmosis, including avirulent strains. In addition, evaluation of lncRNA expression in other human retinal cells that participate in the immune response to *T. gondii*, such as microglia and pigment epithelial cells, could be extremely informative.

## 4. Materials and Methods

### 4.1. Human Subjects

The use of human eyecups for this research was approved by the Southern Adelaide Clinical Human Research Ethics Committee (protocol number: 175.13). Three human cadaver donor eyecup pairs were obtained from the Eye Bank of South Australia (Adelaide, Australia). The three donors were aged 62 to 71 years at death, and the time from death to processing of the eyecups averaged 11 h.

### 4.2. Cell Culture

The MIO-M1 (Moorfields Eye Hospital/University College London Institute of Ophthalmology—Müller 1) cell line (kind gift of G. Astrid Limb, PhD, and Peng T. Khaw, MD, PhD, University College London, London, UK) [62] was cultured in Dulbecco’s modified Eagle’s medium (DMEM; Thermo Fisher Scientific-GIBCO, Grand Island, NY, USA) with 10% heat-inactivated fetal bovine serum (FBS; Bovogen Biologicals, Keilor East, Australia or Thermo Fisher Scientific-GIBCO) at 37 °C and 5% CO_2_ in air.

Retinae from donor eyecup pairs were individually digested in 0.5 mg/mL of Dispase II in phosphate-buffered saline (PBS) with 2% FBS for 10 min at 37 °C. Digested tissue was dissociated by pipetting, passed through a 30 μm strainer, and pelleted by centrifugation. Cells were resuspended in DMEM, Non-Essential Amino Acids Solution, GlutaMAX Supplement, 1 mM sodium pyruvate, 100 U/mL penicillin–100 μg/mL streptomycin (all from Thermo Fisher Scientific-GIBCO), and 10% FBS, plated into 10 cm diameter dishes, and incubated at 37 °C and 5% CO_2_ in air. Half the medium was changed after 5 days in culture and then once per week.

**Figure 5:**
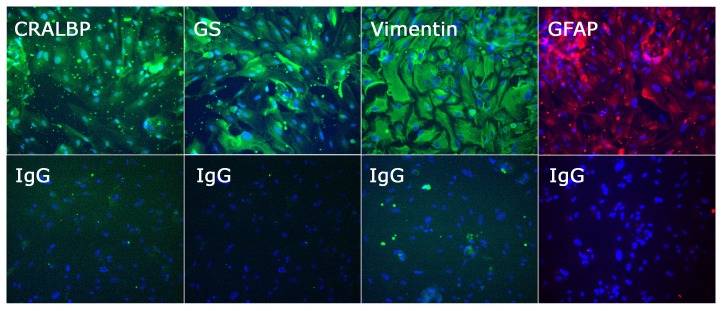
Photomicrographs of Müller cells isolated from human retinae and phenotyped by immunocytochemistry for cellular retinaldehyde binding protein (CRALBP), glutamine synthetase (GS), vimentin, and glial fibrillary acidic protein (GFAP), with negative control labelled with species-matched primary antibody (IgG). Cells stained positively for CRALBP, GS, vimentin, and GFAP; staining artefacts were visible in the negative control, but the labelling pattern was clearly different. Alexa Fluor 488 (green) or Alexa Fluor 594 (red) with DAPI nuclear counterstain (blue). Original magnification: 200×.

The phenotype of retinal cells isolated by this method was assessed on expression of Müller markers: cellular retinaldehyde binding protein (CRALBP), glutamine synthetase (GS), vimentin, and glial fibrillary acidic protein (GFAP), as presented in Figure 5. Cells were fixed with 4% of paraformaldehyde for 10 min, permeabilized with 0.2% Triton X-100 for 5 min, and blocked for 1 h with 2% bovine serum albumin (BSA) and 0.05% Triton X-100 in PBS at room temperature. Cells were incubated overnight at 4 °C with primary antibody diluted to working concentration in 2% BSA–0.05% Triton X-100 in PBS (Supplementary Table S1), subsequently with Alexa 488- or 594-conjugated secondary antibody for 1 h at room temperature, and finally with 4′,6-diamidino-2-phenylindole stain for 2 min. Between steps, cells were washed three times with 0.1% Tween in PBS for 5 min. Immunolabelled cells were imaged on the IX53 Inverted Microscope (Olympus, Tokyo, Japan).

### 4.3. Parasite Culture

Natural isolate *T. gondii* strains were maintained in tachyzoite form by serial passage in confluent human dermal fibroblast monolayers (Cascade Biologics, Portland, OR, USA) in DMEM supplemented with 1% heat-inactivated FBS at 37 °C and 5% CO_2_ in air—GT1 (type I, haplogroup 1) and GPHT (haplogroup 6) (kind gifts of Dr. Jitender P. Dubey, U.S. Department of Agriculture, Beltsville, MD, and Dr. L. David Sibley, Washington University, St Louis, MO, USA) [63,64]. Parasite viability was evaluated by plaque assay in fibroblast monolayers, and required to be at least 15%, which is the expected viability of freshly egressed tachyzoites [65].

### 4.4. Infection of MIO-M1 Cells

Confluent monolayers of MIO-M1 cells in 6-well plates were infected with freshly egressed GT1 or GPHT *T. gondii* tachyzoites in DMEM with 1% heat-inactivated FCS at a multiplicity of infection of 5, or treated with fresh medium alone (*n* = 3 wells per condition). Cell monolayers were incubated at 37 °C and 5% CO_2_ in air, and harvested at 4, 12, 24, and 36 h post-infection.

### 4.5. RNA Isolation and Reverse Transcription

Total RNA was extracted from primary retinal Müller cells and MIO-M1 cells with TRIzol Reagent (Thermo Fisher Scientific-Ambion, Carlsbad, CA, USA), used according to the retailer’s instructions, and frozen at −80 °C. RNA concentration was determined by spectrophotometry on the NanoDrop 2000 (Thermo Fisher Scientific, Wilmington, DE, USA). Reverse transcription was performed using the iScript Reverse Transcription Supermix for RT-qPCR (Bio-Rad, Hercules, CA, USA), with 100 ng (PCR) or 500 ng (qPCR) of RNA template yielding 20 μL cDNA.

### 4.6. Polymerase Chain Reaction

PCR was performed on the T100 Thermocycler (Bio-Rad) using 1.5 μL of cDNA diluted to 1:5, 2.5 μL of 10× PCR buffer (Bio-Rad), 0.5 μL of 10 mM dNTP mix, 1.2 μL of 25 mM magnesium chloride, 1.25 μL each of 20 μM forward and reverse primers (Supplementary Table S2), 0.2 μL of HotStar Taq Polymerase, and 16.6 μL of nuclease-free water for each reaction. Amplification consisted of a pre-cycling hold at 95 °C for 5 min, 40 cycles of denaturation for 30 s at 95 °C, annealing for 30 s at 60 °C (or 58.2 °C for SNHG15, or 63.3 °C for MIR143HG), extension for 1 min at 72 °C, and a post-extension hold at 72 °C for 5 min. Sizes of PCR products were confirmed by electrophoresis with SYBR Safe DNA Gel Stain (Thermo Fisher Scientific-Invitrogen, Carlsbad, CA, USA) on 2% or 3% agarose gel, depending on the expected product size.

### 4.7. Quantitative Real-Time Polymerase Chain Reaction

The RT-qPCR was performed on the CFX Connect Real-Time PCR Detection System (Bio-Rad) using 2 μL of cDNA diluted to 1:10, 4 μL of iQ SYBR Green Supermix (Bio-Rad), 1.5 μL each of 20 μM forward and reverse primers (Supplementary Table S2), and 11 μL of nuclease-free water for each reaction. Amplification consisted of a pre-cycling hold at 95 °C for 5 min, 40 cycles of denaturation for 30 s at 95 °C, annealing for 30 s at 60 °C, extension for 30 s at 72 °C, and a post-extension hold at 75 °C for 1 s. A melting curve, representing a 1 s hold at every 0.5 °C between 70 °C and 95 °C, was generated to confirm that a single peak was produced for each primer set. The cycle threshold was measured, with Cq determination mode set to regression. Relative expression was determined using the Pfaffl mathematical model [66], and normalized to two stable reference genes: ribosomal protein lateral stalk subunit P0 (RPLP0) and peptidylprolyl isomerase A (PPIA). Stability of reference gene expression was assessed using the method described by Hellemans et al. [67], which we have used in previous work [68]; this involves calculation of the gene-stability measure (M-value) and the coefficient of variance (CV-value), and requires an M-value of less than 0.5 and a CV-value of less than 0.25. These calculations were performed with the Gene Expression Analysis module of CFX Manager v3.1 software (Bio-Rad). Transcript expression in *T. gondii*-infected and uninfected MIO-M1 cells were compared by two-way ANOVA with Bonferroni post-test. A *p*-value less than 0.05 was taken to indicate statistical significance.

## Supplementary Materials

The following are available online at https://www.mdpi.com/2311-553X/5/4/48/s1, Table S1: Primary antibodies for retinal Müller cell markers; Table S2: Primer sequences and product sizes for gene transcripts.
