# Supplementary material for: Expression of Long Non-Coding RNAs by Human Retinal Müller Glial Cells Infected with Clonal and Exotic Virulent Toxoplasma gondii
Source: Noncoding RNA. 2019 Sep 20;5(4):48. doi: 10.3390/ncrna5040048 (PMC6958423; doi:10.3390/ncrna5040048)
Supplement: Supplementary file 1 [file ncrna-05-00048-s001.zip › SupplementaryTable1.FINAL.docx]

**Supplementary Table 1.** Primary antibodies for retinal Müller cell markers.

| **Target** | **Host** | **Supplier** | **Working concentration**  **(μg/mL) or dilution** |
| --- | --- | --- | --- |
| Vimentin (MAB3400) | Mouse | Merck Millipore | 5 |
| CRALBP (PA5-29759) | Rabbit | Thermo Fisher Scientific | 2 |
| GS (G2781) | Rabbit | Merck Sigma-Aldrich | 10 |
| GFAP (AF2594) | Sheep | R&D Systems | 1:125 |
