# Supplementary material for: Expression of Long Non-Coding RNAs by Human Retinal Müller Glial Cells Infected with Clonal and Exotic Virulent Toxoplasma gondii
Source: Noncoding RNA. 2019 Sep 20;5(4):48. doi: 10.3390/ncrna5040048 (PMC6958423; doi:10.3390/ncrna5040048)
Supplement: Supplementary file 1 [file ncrna-05-00048-s001.zip › SupplementaryTable2.FINAL.docx]

**Supplementary Table 2.** Primer sequences and product sizes for gene transcripts. Sequences were either sourced from the published literature (indicated by asterisk) or designed in-house.

| **Gene transcript** | **Primer pair** | **Product size (bp)** |
| --- | --- | --- |
| APTR***** | Forward 5’- TGTGGGTACAAAAGGAGAGTAACAT-3’  Reverse 5’- GTAGATCTGGAGCTGCAACTACAG-3’ | 300 |
| BANCR***** | Forward 5’- ACAGGACTCCATGGCAAACG -3’  Reverse 5’- ATGAAGAAAGCCTGGTGCAGT -3’ | 80 |
| CDKN2B-AS1***** | Forward 5’- AACCGGGGAGATCTATTTGG-3’  Reverse 5’- GGTGTGGTGTCTCACACCTG-3’ | 169 |
| CYTOR***** | Forward 5’- ACCGAAAATCACGACTCAGCCC-3’  Reverse 5’- AATGGGAAACCGACCAGACCAG-3’ | 187 |
| FIRRE***** | Forward 5’- CTGTGACCTCGCTTCACTTCT -3’  Reverse 5’- GTGGCAAAGAGCAGAAGATAGA -3’ | 215 |
| FOXD3-AS1***** | Forward 5’- GGTGGAGGAGGCGAGGATG -3’  Reverse 5’- AGCGGACAGACAGGGATTGG -3’ | 118 |
| GAS5***** | Forward 5’- TCTTGCCTCACCCAAGCTAGAG-3’  Reverse 5’- TTGTGCCATGAGACTCCATCAG-3’ | 127 |
| GSTT1-AS1***** | Forward 5’- CTTTTGCATAGAGACCATGACCAG-3’  Reverse 5’- TGGATAATAAACCTGGGCTCAGC-3’ | 105 |
| HOTAIR***** | Forward 5’- GGTAGAAAAAGCAACCACGAAGC -3’  Reverse 5’- ACATAAACCTCTGTCTGTGAGTGCC -3’ | 168 |
| HoxD-AS1***** | Forward 5’- TCTGAAAGAAGGACCAAAGTAA -3’  Reverse 5’- ATTCAAGGGACAGTCACAGG -3’ | 149 |
| KCNQ1OT1***** | Forward 5’- AATATGGATTCCTAACTGAGCCC-3’  Reverse 5’- TGCCTTCTGCCAACACTTGGC-3’ | 567 |
| LINC00305***** | Forward 5’- TCAGCAGCCTTCTGGTTTATCA-3’  Reverse 5’- TCCTTGCTTCCTTCAGGTCTCT -3’ | 162 |
| LINC00968 | Forward 5’-AATTGTGTCCCCTGTCCACC-3’  Reverse 5’-CTGTGCTGAGCTGTCTGGAA-3’ | 126 |
| LINC01105 | Forward 5’-AGG AGA AGG AAT TGT GGC GG-3’  Reverse 5’-CAG TCC CTG CTG TAG CGA AA-3’ | 137 |
| LINC-ROR***** | Forward 5’- GCCTGAGAGTTGGCATGAAT-3’  Reverse 5’- AAAACCTCACTCCCATGTGC -3’ | 151 |
| lnc-SGK1***** | Forward 5’- AGAGGACGCAGGAGATTGGA -3’  Reverse 5’- CAAGGCTGAAGCATCTCCGTA-3’ | 131 |
| LUCAT1***** | Forward 5’- GCTCGGATTGCCTTAGACAG-3’  Reverse 5’- GGGTGAGCTTCTTGTGAGGA-3’ | 114 |
| MALAT1 | Forward 5’- ATT CCG GTG ATG CGA GTT GT -3’  Reverse 5’- ATT CGG GGC TCT GTA GTC CT -3’ | 396 |
| MEG3 | Forward 5’- GAGTGTTTCCCTCCCCAAGG-3’  Reverse 5’- GCGTGCCTTTGGTGATTAG-3’ | 187 |
| MHRT***** | Forward 5’- CCGACTGCGACTCCTCATAC-3’  Reverse 5’- GGCTGAAGAGTGAGCCTTGT-3’ | 73 |
| MIAT***** | Forward 5’- GCTGACCACTAACAACCAACC -3’  Reverse 5’- AGGAACAGACCAGGAAGGCAG -3’ | 122 |
| MIR17HG***** | Forward 5’- TGATGGTGGCCTGCTATTTC-3’  Reverse 5’- ACAGTTTGATTGGGCGACAG-3’ | 221 |
| MIR22HG***** | Forward 5’- CGGACGCAGTGATTTGCT -3’  Reverse 5’- GCTTTAGCTGGGTCAGGACA -3’ | 136/219 |
| MIR143HG***** (PCR) | Forward 5’- CCAACCACTCCCCAAACA-3’  Reverse 5’- TTACAGCCGTTGCTCTCCTT-3’ | 128 |
| MIR143HG***** (qPCR) | Forward 5’- AGGGCCAGCAGCAGGC-3’  Reverse 5’- TCAGGAAATGTCTCTGGCTGTG-3’ | 101 |
| MIR155HG***** | Forward 5’-GAGTGCTGAAGGCTTGCTGT-3’  Reverse 5’-TTGAACATCCCAGTGACCAG-3’ | 247 |
| NEAT1 | Forward 5’- CTTGGCACTGGTACTGGGAG -3’  Reverse 5’- ACCCACGCACTAAATTCCCC -3’ | 137 |
| NeST***** | Forward 5’- AGGAAGCTGGGTAATTGAATGC -3’  Reverse 5’- CTTAGGAGGAGAATTTTGGGAGAG -3’ | 94 |
| NRIR***** | Forward 5’- CCACCCCCACGAAGAAATTATATATC-3’  Reverse 5’- GTTAGAGGTGTCTGCTGCAATAATC-3’ | 247 |
| NRON***** | Forward 5’- ACGTTCCTTAATGTACGCCTTTGC-3’  Reverse 5’- TTGGCCGTGTCCTGAGTCCTT-3’ | 133 |
| PACER***** | Forward 5’- TGTAAATAGTTAATGTGAGCTCCACG-3’  Reverse 5’- GCAAATTCTGGCCATCGC -3’ | 90 |
| PPIA | Forward 5’-GAG CAC TGG AGA GAA AGG ATT T-3’  Reverse 5’-GGT GAT CTT CTT GCT GGT CTT-3’ | 355 |
| PURPL***** | Forward 5’- CGTGTGAAAAGAACCCAGGTA -3’  Reverse 5’- CGCCTGGTAAAACAACCAGT -3’ | 120 |
| PVT1***** | Forward 5’- AGCACTCTGGACGGACTTGAGA-3’  Reverse 5’- CCACTAGCAGCAACAGGAGAAG-3’ | 179 |
| RMRP***** | Forward 5’- GAGAGTGCCACGTGCATACG-3’  Reverse 5’- ACGCTTCTTGGCGGACTTT-3’ | 67 |
| RPLP0 | Forward 5’-GCA GCA TCT ACA ACC CTG AA-3’  Reverse 5’-GCA GAT GGA TCA GCC AAG AA-3’ | 235 |
| SAG1 | Forward 5’- GCTGTAACATTGAGCTCCTTGASTTCCTG-3’  Reverse 5’- CCGGAACAGTACTGATTGTTGTCTTGAG-3’ | 355 |
| SNHG15***** | Forward 5’- GCTGAGGTGACGGTCTCAAA-3’  Reverse 5’- GCCTCCCAGTTTCATGGACA-3’ | 155 |
| SOCS2-AS1***** | Forward 5’- CCATACAGGTCAACTTTTCCACCAC -3’  Reverse 5’- CCAACCTCAGCTCTGCTCTCTT -3’ | 83 |

Abbreviations: APTR= alu-mediated CDKN1A/P21 transcriptional regulator; BANCR= BRAF-activated non-protein coding RNA; CDKN2-AS1= cyclin-dependent kinase Inhibitor 2A - antisense RNA; CYTOR= cytoskeleton regulator RNA (LINC00152); FIRRE= functional intergenic repeating RNA element; FOXD3-AS1= forkhead box D3 - antisense RNA 1; GAS5= growth arrest specific 5; GSTT1-AS1= glutathione S-transferase theta 1 antisense RNA 1 (lncRNA-CD244); HOTAIR= HOX transcript antisense RNA; HoxD-AS1= HOXD cluster antisense RNA 1; KCNQ1OT1= KCNQ1 opposite strand/antisense transcript 1; LINC= long intergenic non-protein coding RNA; LINC-ROR= LINC regulator of reprogramming; lnc-SGK1= long non-coding serum and glucocorticoid-inducible kinase; LUCAT1= lung cancer associated transcript 1; MALAT1= metastasis associated lung adenocarcinoma transcript 1; MEG3= maternally expressed 3; MHRT= myosin heavy chain associated RNA transcripts; MIAT= myocardial infarction associated transcript (Gomafu); MIR17HG= MIR17 host gene; MIR22HG= MIR22 host gene; MIR143HG= MIR143 host gene (CARMN); MIR155HG= MIR155 host gene; NEAT1= nuclear paraspeckle assembly transcript 1; NeST= nettoie salmonella pas Theiler’s (= Tmevpg1= Theiler's murine encephalomyelitis virus persistence candidate gene 1= IfngAS1); NRIR= LncRNA-CMPK2= negative regulator of interferon response; NRON= non-coding repressor of NFAT; PPIA= peptidylprolyl isomerase A; RPLP0= ribosomal protein lateral stalk subunit P0; PACER= PTGS2 antisense NFKB1 complex-mediated expression regulator RNA; PURPL= p53 upregulated regulator of p53 levels; PVT1= plasmacytoma variant translocation 1; RMRP= RNA component of mitochondrial RNA processing endoribonuclease; SAG1= surface antigen 1; SNHG15= small nucleolar RNA host gene 15; SOCS2-AS1= suppressor of cytokine signaling 2 - antisense RNA 1.
